# Magnitude and distribution of stresses in composite resin and sound dentine interface with mechanical retentions

**DOI:** 10.4317/jced.52144

**Published:** 2015-04-01

**Authors:** Gisaku Kuramochi, Eduardo Borie, Iara-Augusta Orsi, Mariano Del Sol

**Affiliations:** 1 Department of Restorative Dentistry, School of Dentistry, Universidad Finis Terrae, Av. Pedro de Valdivia 1509, 7501015 Santiago, Chile; 2 PhD, Student of Doctoral Program in Medical Sciences, Medicine School, Manuel Montt 112, 4781176 Universidad de La Frontera, Temuco, Chile; 3 CIMOFIR, Research Centre, Dental School, Universidad de La Frontera, Manuel Montt 112, 4781176 Temuco, Chile; 4 Dental Materials and Prosthodontics Department, Ribeirão Preto Dental School, University of São Paulo, Av. do Café w/n, 14040904 Ribeirão Preto, SP, Brazil; 5 Department of Basic Sciences, Faculty of Medicine, Universidad de La Frontera, Av. Francisco Salazar 01145, 4811230 Temuco, Chile

## Abstract

**Background:**

Adhesive systems are constantly subjected to mechanical and chemical stresses that negatively impact the integrity and durability of the dentine-adhesive interface. Despite the lack of evidence to support or reject the clinical indication for mechanical retention, the potential further contribution of these preparations to the behavior of the composite resin-sound dentine bond has been rarely addressed. The authors evaluated by finite element analysis the effect of mechanical retention on the magnitude and distribution of stresses in a composite resin-sound dentin bonding interface when subjected to tensile and shear forces.

**Material and Methods:**

A three-dimensional model was created based on three cylindrical volumes representing the sound dentin, adhesive system, and composite resin. From this main model, two models were designed to simulate dentine bonding: 1) a model with no mechanical retention, which considered flat adhesion; and 2) a model with retention, which considered four hemispherical holes on the dentine surface. Both groups were subjected to linear static analysis under tensile and shear loading of 200N.

**Results:**

At the model with retentions’ bonding interface under tensile and shear loading, a concentration of Von Mises equivalent stress was observed within the retentions, with a reduction of those stresses on the bonding boundary surface.

**Conclusions:**

Additional mechanical retention increases the tensile strength of the sound dentin-composite resin bonding interface, promoting a decrease in the magnitude of the stresses and their redistribution under tensile and shear loading.

** Key words:**Adhesion, composite resins, dentine, finite element analysis.

## Introduction

The adhesion of composites to dental tissues has proven to be a successful technique for dental restoration when teeth are affected by decay or trauma (1). The lack of retention does not seem to be the main cause of the premature loss of bonded restorations (2). The survival of these restorations and their adhesion values differ depending on the tissue involved and its location (3). This difference is especially critical at the level of exposed cervical dentine due to stress concentration (4) and to changes in the nature of the substrate over time (5), as its adhesion values are lower compared to enamel and sound dentine (6).

Adhesive systems are constantly subjected to mechanical and chemical stresses that negatively impact the integrity and durability of the dentine-adhesive interface (4,7). Despite the progress made in the development of new adhesive systems and strategies to overcome these limitations (8,9), our knowledge of the behavior of the interface over time remains limited. In fact, high variability is found among the results reported by different laboratory studies (8,9), and their correlation with the clinical behavior of cervical restorations has not been sufficiently established (10).

The presence of exposed dentine is generally associated with a set of lesions known as non-carious cervical lesions (NCCL) (11). Currently, their prevalence is increasing, and they occur in direct relationship with age (12). Recently, survival studies in class V restorations identified certain risk factors strongly associated with the early loss of NCCL restorations (13), one of which is cavity preparation design, which in turn must consider not only the type of substrate but also the stress concentration to which it is subjected. Therefore, some authors as Brackket *et al.* (14) have suggested performing additional mechanical retention.

The rational use of additional mechanical retention to improve resistance at the cervical level could lead to much more conservative and less invasive restorations than the cavity preparation designs suggested for the treatment of class V lesions (15). The same approach could be applied during the replacement of cervical restorations, a procedure that is associated with a significant increase in the size of the original preparation (15).

In general, research has focused primarily on the study of new adhesive systems that overcome the limitations imposed by the substrate (1). However, due to the importance of the biomechanical features involved in the behavior of the bonding interface and the difficulty of determining this behavior clinically, it requires mathematical models able to accurately replicate the clinical reality (16).

Thus, through the use of FEM it is possible to obtain reliable information related to tensile, compressive, and shear stresses, or the combination of these, as a result of the behavior of the model under an applied load (17).

Despite the lack of evidence to support or reject the clinical indication for mechanical retention, the potential further contribution of these preparations to the behavior of the CR-SD bond has been rarely addressed. Thus, the aim of this study was to evaluate by finite element analysis, in three dimensions, the use of hemispherical mechanical retention cut into dentine in relation to the reduction of the magnitude and distribution of stresses at the composite-dentine interface when subjected to tensile and shear forces.

## Material and Methods

-CAD/FEA Model

**Figure 1:**
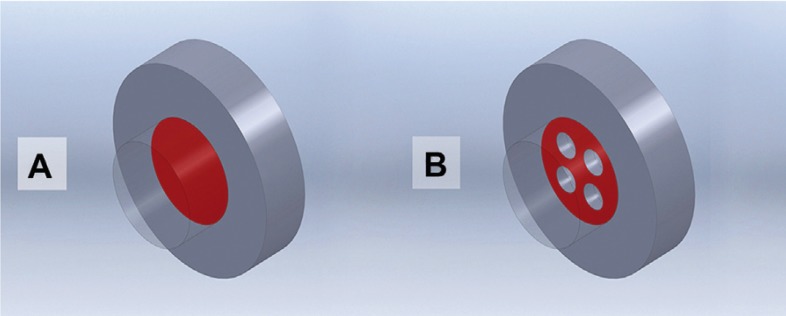
A) Model without retentions (NRM); B) Model with retentions (RM). In red is marked the analyzed area.

A three-dimensional (3D) model was developed to analyze the magnitude and distribution of the shear and tensile stress loads on the CR and dentine-bonding interface when mechanical retentions are cut into the dentine. The analysis was based on a simplified model of 3 cylinder volumes that represented each element as follows: dentine (8 mm in diameter and 2 mm in thickness), adhesive (4 mm in diameter and 10 µm in thickness), and CR (4 mm in diameter and 2 mm in thickness). These models were designed using the software CATIA (V5R16 Dassault Systèmes, Versailles, France) and saved in a neutral format (.stp) for easy export to the analysis software. For our purposes, the analysis was circumscribed to the marked area in the figure 1.

Considering the geometry representing the interface, the following two experimental models were developed to simulate the different adhesion conditions.

- Model with no mechanical retentions (NRM): This model considers only adhesion on a flat surface with no additional mechani-cal retentions (Fig. 1A).

- Model with retention (RM): This model considers in its geometry 4 cylindrical holes in the dentine surface (Fig. 1B), each ending in a hemisphere that is 1 mm in diameter and 1 mm deep, such that the resin penetrates inside of the dentine to generate a RM effect preventing its dislodgement.

The model simulation was performed by exporting the models into the finite element software Ansys Workbench v.14 (Ansys Inc., Canonsburg, PA, USA), and the structures for static analysis were considered isotropic, homogeneous, flexible, and linearly elastic. The elasticity modulus and Poisson coefficient were obtained from the literature (18,19) ( Table 1).

**Table 1:**
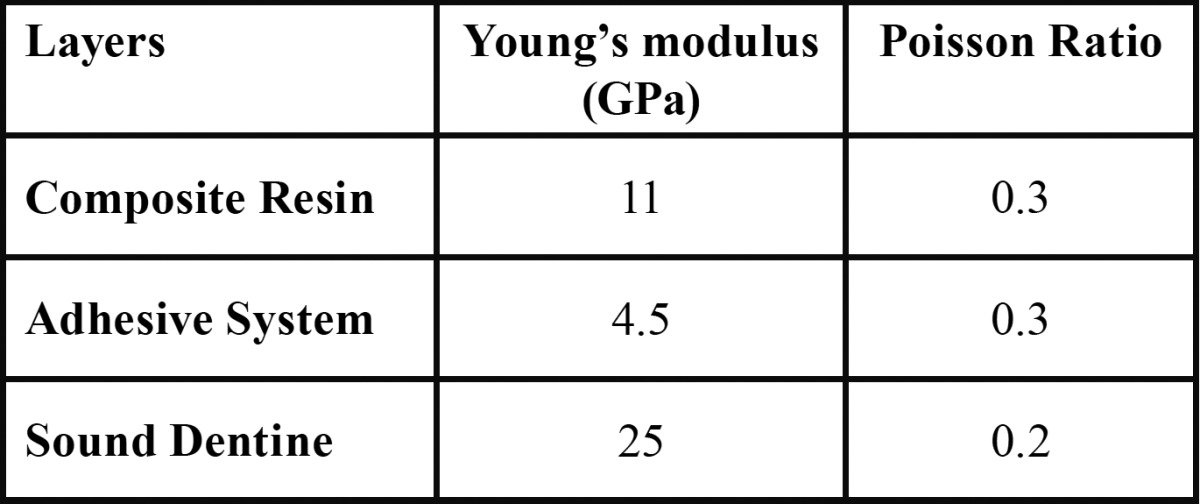
Mechanical properties of each model’s layer.

**Figure 2:**
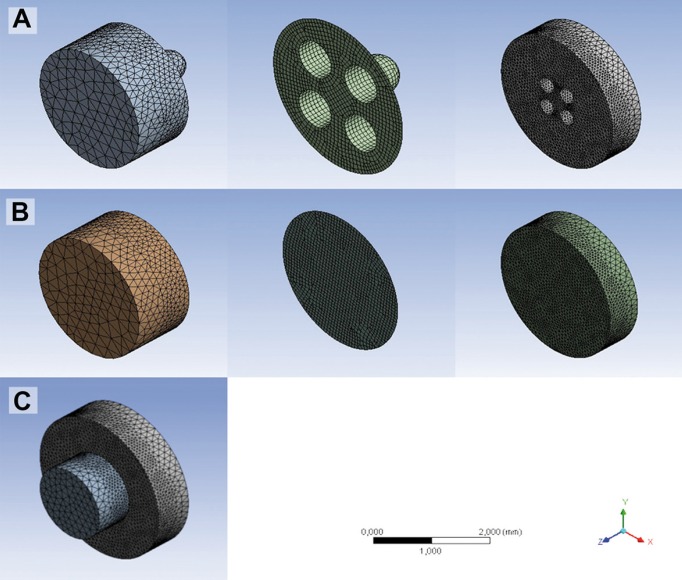
Discretization of the resin-adhesive-dentin layers in A) RM, B) NRM and C) the final assembled model.

Additionally, the solid cylinders of dentine and CR were meshed with SOLID185-type tetrahedral elements and the adhesive surface with quadrilateral elements and triangular SHELL181-like membranes (20) For the RM, 149266 elements and 29497 nodes were obtained; for the NRM, the result was a total of 129258 elements and 24945 nodes. To obtain convergent results, the adhesive surface was refined considering 0.1 mm to be the maximum size of the elements, and the solid components of the dentine and CR were refined in the area near the interface with a maximum element size of 0.2 mm (Fig. 2).

With the aim of understanding the effect of the mechanical retentions on the different elements in the model, the simulation was performed to obtain the stress load generated when the model was subjected to shear and tensile loading. To compare the resulting stresses, a shearing load of 200N obtained from the literature (21) was applied to the composite resin surface at 1 mm of separation from the dentine-CR bond interface in both the RM and NRM; in addition, simulations with a 200N (21) tensile load were performed to test the adhesive capacity. The contacts between dentine-adhesive and adhesive-CR were considered bonded.

The analyses with Von Mises equivalent stress were performed through graphical visualization of colors map to compare the models, describing the images both quantitatively and qualitatively. All graphic scales were standardized.

## Results

-Stresses due to shear load

According to the results obtained from the analysis for the applied load condition, it is possible to find a decrease of Von Mises equivalent stress in the dentine and composite resin of the RM. However, an increase could be observed in the stress on the adhesive layer at the inner of the retention area ( Table 2).

**Table 2:**
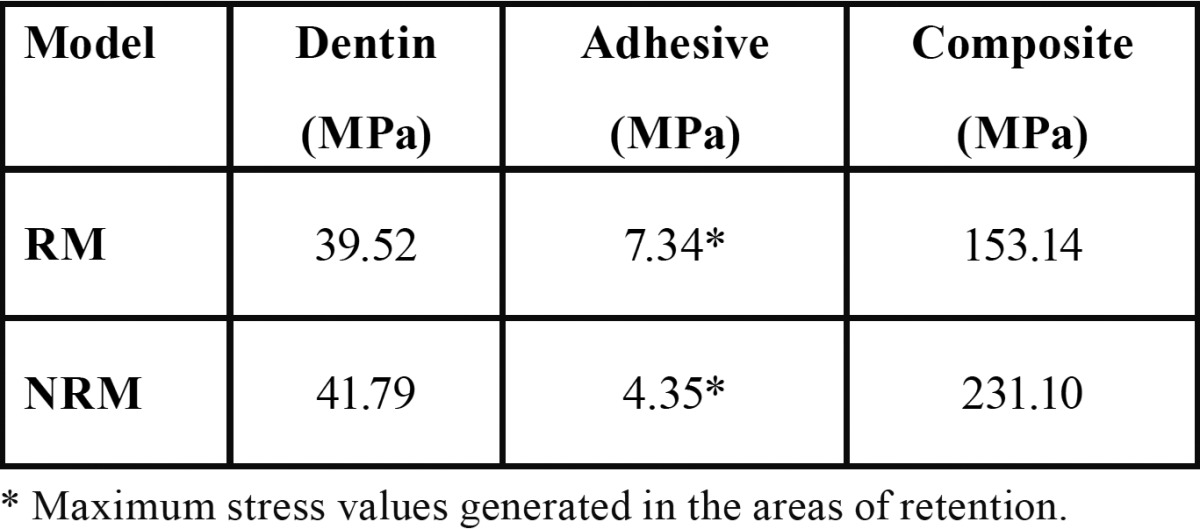
Magnitude of Equivalent Von Mises stress on each layer under shear load.

-Stresses due to tensile load

The Von Mises equivalent stress values generated on the RM when applied tensile load tends to increase in all its components compared to the NRM ( Table 3). Also, it can be observed a stress at the interface of the retention model under shear and tensile loading and its magnitude is expressed in  Table 4.

**Table 3:**
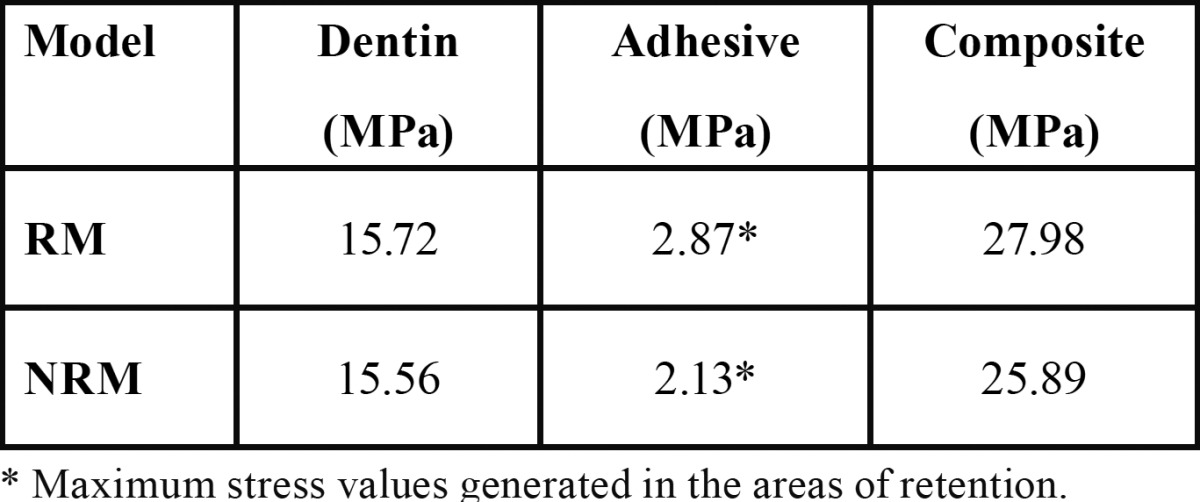
Magnitude of Equivalent Von Mises stress on each layer under tensile load.

**Table 4:**
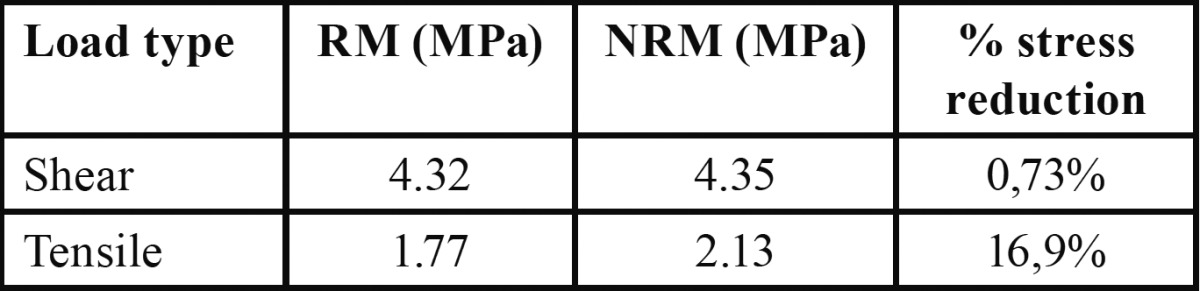
Equivalent Von Mises stress values at the interface in both models.

**Figure 3:**
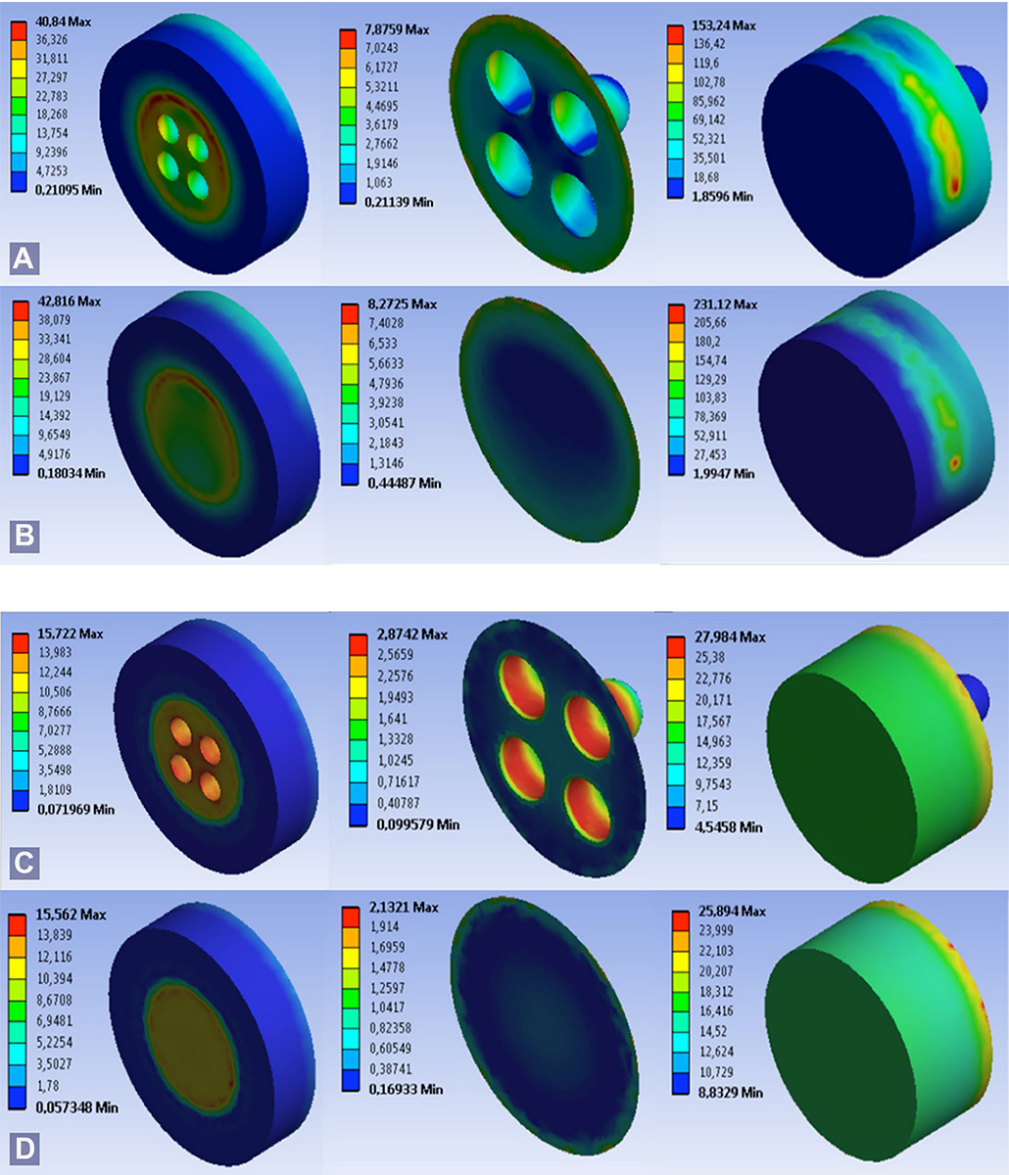
Distribution of equivalent Von Mises stress the dentin, adhesive and resin layers under shear load A) RM; B) NRM and under tensile load C) RM; D) NRM.

Additionally, under tensile and shear loading in the RM, a redistribution of the maximum stresses was identified at the bonding interface, decreasing on the surface of the adhesion area and concentrating in the interior of retentions (Fig. 3). In the NRM, the maximum interfacial stresses were concentrated at the boundary of the adhesion area.

Nonetheless, under tensile loading, a decrease in maximum stresses is also observed at the interface of the adhesion area in the RM, where stresses also decrease on the load-bearing adhesive plane (Fig. 3).

## Discussion

While the current state of development allows the use of adhesive restorative materials without mechanical retention as a primary source of adhesion, the continued integrity of the bonding interface over time is affected by, among other factors, the distribution of the stresses to which it is subjected (4). Some authors have suggested the use of additional mechanical retention, especially in the areas of greatest stress concentration and low adhesion (22). However, the role of mechanical retention in dentine has not been adequately studied in relation to any adhesive system.

 As a first approach in the search for evidence, we have resorted to the use of FEM, which has proven to be a suitable method of performing clear and objective tests on the biological systems proposed as the object of study (17,23). Our study shows that when shear loading is applied on the RM, the equivalent Von Mises stress decreased in both composite resin and dentine; however, they increase in the adhesive that covers the interior of the retentions, which may be due in part to the material change caused by including retentions.

However, in the same model under tensile loading, the equivalent Von Mises stress increased in all the layers, which could be explained by the increased contact area provided by the holes. Likewise, a decrease in the stresses was observed at the RM inter-face for both types of loading, which could also be explained by the above factors.

Despite the results observed in this study, Heintze and Rousson (24) argue that the tensile test has greater clinical significance, although the value of tensile strength at the interface obtained in our study (16.9%) is interesting. Recent long-term clinical studies of old adhesive systems with much lower adhesion values than current systems revealed high restoration longevity (13). Therefore, obtaining higher adhesion force in the adhesive system does not seem to be the primary factor in good clinical performance over time.

Interestingly, with both types of loading, a redistribution of stresses in the adhesion area can be observed, concentrated within the mechanical retentions cut in the dentine. In this way, the remaining load-bearing bonding interface area increases in strength and reduces the stress concentration at the margins. This development would preserve the integrity of the interface, decreasing the tendency to adhesion failure (7), and simultaneously represent a contribution in obtaining and maintaining a good marginal seal, which would improve the long-term performance of restorations by minimizing microleakage and its consequences, such as postoperative sensitivity, discoloration, pulp inflammation, and caries (2).

The results obtained through FEM in this study revealed that mechanical retention favors a redistribution of such stresses and a decrease of their magnitude in the bonding interface, thereby helping to maintain integrity. However, these results should be treated with caution due to the inherent limitations of the models. Several factors can affect dentine adhesion, including the sensitivity of the technique and the degree of dentinal mineralization and sclerosis (25), the surface of which can present a very different composition from sound dentine (26).

Although cervical lesions in the mouth present much more complex configurations that vary in the form and amount of tissue involved, in this study the design of a single flat surface model for the simulations was intended to facilitate the analysis and interpretation of findings. Therefore, it would be interesting to obtain further evidence regarding the behavior of the bonding interface when a RM is prepared. Obtaining such evidence would require considering other designs, incorporating various approaches, and using non-linear analysis to more accurately replicate clinical reality (16).

Even so, the convenience of using additional retentions involves first addressing certain aspects such as postoperative sensitivity, component integrity, and the functional- biological preservation of the pulp; the findings of such studies would improve our knowledge of the optimal number and configuration for the preservation of both the structural and biological integrity of the tissues involved and the stability of the interface. Only then will it be possible to better judge the relevance of their incorporation in the treatment of cervical lesions in exposed dentine.

Within the limitations of the study, it can be concluded that mechanical retention seems to favor a redistribution of such stresses and a decrease of their magnitude in the bonding interface, thereby helping to maintain the integrity.
